# Symptomless turnip yellows virus infection causes grain yield loss in lentil and field pea: A three-year field study in south-eastern Australia

**DOI:** 10.3389/fpls.2022.1049905

**Published:** 2022-11-23

**Authors:** Narelle Nancarrow, Mohammad Aftab, Grant Hollaway, Brendan Rodoni, Piotr Trębicki

**Affiliations:** ^1^ Agriculture Victoria, Grains Innovation Park, Horsham, VIC, Australia; ^2^ Agriculture Victoria, AgriBio Centre, Bundoora, VIC, Australia; ^3^ School of Applied Systems Biology, La Trobe University, Bundoora, VIC, Australia; ^4^ School of Agriculture and Food, The University of Melbourne, Parkville, VIC, Australia

**Keywords:** TuYV, *Lens culinaris*, *Pisum sativum*, yield loss, aphids, *Myzus persicae*, symptomless plant virus infection

## Abstract

*Turnip yellows virus* (TuYV) is a damaging virus that is persistently transmitted by aphids and infects a wide range of grain hosts including lentil (*Lens culinaris* Medik), field pea (*Pisum sativum* L.) and canola (*Brassica napus* L., oilseed rape). Although information is available about the effects of TuYV infection on grain yield in canola, data about its impact on yield in pulses is lacking. In this study, field experiments quantifying the effects of TuYV infection on the grain yield of lentil and field pea were conducted over three consecutive years (2018-2020) with varying weather conditions. Plants artificially inoculated with TuYV using viruliferous green peach aphid (*Myzus persicae*, Sulzer) were grown under typical field conditions in south-eastern Australia. At maturity, grain yield, along with associated grain and plant growth parameters, were measured. Compared to the non-inoculated control treatment, early TuYV infection reduced grain yield by up to 36% in lentil and 45% in field pea, while late TuYV infection had no significant impact on yield. Despite a high incidence of TuYV infection and significant yield losses recorded in inoculated plots, no obvious symptoms of virus infection were observed in the inoculated plots in any of the six experiments; this lack of visible symptoms in lentil and field pea has significant implications for crop health assessments, demonstrating the importance of testing for virus instead of relying solely on the presence of visual symptoms, and may also be leading to an underestimation of the importance of TuYV in pulses in Australia.

## Introduction

Pulses such as chickpea (*Cicer arietinum* L.), faba bean (*Vicia faba* L.), field pea (*Pisum sativum* L.), lentil (*Lens culinaris* Medik), and lupin (*Lupinus angustifolius* L. and *L. albus* L.) are grain legumes that are grown globally for human or animal consumption. In Australia, these pulses are grown throughout the winter months where they are typically sown from May-June and harvested from November-January. They are grown in rotation with cereals and canola due to their ability to fix atmospheric nitrogen (Beckie and Brandt, 1997; Campbell et al., 2007), and as a break crop to disrupt disease cycles (Stevenson and van Kessel, 1996; Beckie and Brandt, 1997; Kirkegaard et al., 2008). Approximately 90 million tonnes of pulses were produced globally in 2020 (Statista, 2022) while in Australia, an average of 2.8 million tonnes of pulses, consisting of 835 kt of chickpeas, 782 kt of lupins, 548 kt of lentils, 391 kt of faba beans and 279 kt of field peas, were produced annually from 2016 to 2020 (ABARES, 2021).

Turnip yellows virus (TuYV) is distributed worldwide and has been reported in countries including the United Kingdom (Gilligan et al., 1980; Fowkes et al., 2021), the United States (Duffus and Russell, 1970; Duffus and Russell, 1972), Germany (Schröder, 1994; Gaafar and Ziebell, 2019) and Australia (Coutts et al., 2006; Filardo et al., 2021). TuYV has a wide host range which includes, among others, a variety of pulses, canola (*Brassica napus*, oilseed rape), and weed and pasture hosts (Stevens et al., 1994; Graichen and Rabenstein, 1996; Coutts et al., 2006). TuYV belongs to the genus *Polerovirus* and the family *Solemoviridae* (Mayo, 2002; Scheets et al., 2020) and was originally classified as a European strain of beet western yellows virus (BWYV) (Duffus and Russell, 1970), but was later re-classified as a distinct species (Mayo, 2002). TuYV is a phloem-limited virus that is persistently transmitted by a range of aphid species (Duffus and Russell, 1972; Schliephake et al., 2000), with *Myzus persicae* (Sulzer) (Hemiptera: Aphididae) (the green peach aphid) identified as its most efficient and important vector (Johnstone and Duffus, 1984; Schliephake et al., 2000; Stevens et al., 2008). Typical symptoms of TuYV infection include yellowing or reddening of leaves and/or whole plants, poor or stunted growth, and plants can appear as ‘spindly’, however symptomless infection has also been observed (Jones et al., 2007; Stevens et al., 2008; Schwinghamer et al., 2009; Coutts et al., 2010; Jones et al., 2021).

In Australia, TuYV was first reported as beet western yellows virus (BWYV) in potatoes in Tasmania in 1982 (Duffus and Johnstone, 1982; Jones et al., 2021). TuYV is one of the most widespread viruses affecting pulses in Australia (Freeman et al., 2013; van Leur et al., 2013; Filardo et al., 2021; Jones et al., 2021) and is commonly found in field pea, faba bean, lupin, chickpea and lentil, along with canola and weeds such as wild radish (*Raphanus raphinistrum* L.) and marshmallow (*Malva parviflora* L.) located within and nearby to crops (Johnstone and Duffus, 1984; Coutts and Jones, 2000; Latham and Jones, 2001; Hawkes et al., 2002; Freeman and Aftab, 2011; van Leur et al., 2013; Filardo et al., 2021). In canola, grain yield losses of up to 46% resulting from TuYV infection have been reported in Australia, and it has been estimated that up to 12 kg/ha of yield is lost for each 1% of TuYV incidence (Jones et al., 2007), however, the effect of TuYV infection on yield in pulses is yet to be quantified. To begin addressing this knowledge gap, six field experiments were conducted over three years (2018-2020) with varying weather conditions to quantify the impact of early and late TuYV infection on the grain yield of lentil and field pea in south-eastern Australia.

## Materials and methods

### Field sites and experiments

Field experiments conducted to evaluate the effects of TuYV infection on grain yield and plant growth of lentil (variety PBA Hallmark XT) and field pea (variety PBA Wharton) were grown under typical field conditions in Victoria, Australia. PBA Hallmark XT is a high-yielding red lentil variety while PBA Wharton is a high-yielding field pea variety with some resistance to pea seed-borne mosaic virus and bean leaf roll virus. All experiments were conducted at Agriculture Victoria’s Wimmera Research Station at Longerenong (36°40’S, 142°18’E) in western Victoria, Australia, where the long term (1961-2020) mean annual maximum temperature was 21.6°C and the mean annual rainfall was 403 mm (www.bom.gov.au; www.longpaddock.qld.gov.au/silo, accessed on 15^th^ June 2021) (Jeffrey et al., 2001). Each experiment was direct seeded using a 6- row plot seeder (PJ Green, Grovedale, Australia) into 10 m x 1.8 m bays consisting of six rows with 25 cm spacing, 30 cm between bays and an establishment density target of 120 plants/m^2^ for lentil and 40 plants/m^2^ for field pea. Field experiments were maintained using agronomic practices typical for the area, although insecticide was only included after all inoculation treatments had been applied each year.

### Virus propagation and aphid colony

The virus isolate TuYV-5414 (GenBank accession number MT586591) used in the field experiments was obtained from a naturally infected canola plant displaying typical yellow and red/purple leaf symptoms of TuYV infection in Deniliquin, New South Wales (NSW), Australia during 2015. The virus identity was confirmed using tissue blot immunoassay (TBIA), RT-PCR and high-throughput sequencing (Filardo et al., 2021). Viruliferous *Myzus persicae* were routinely maintained on TuYV-infected canola plants in insect-proof cages in a glasshouse set at 22°C. Aphids were reared on TuYV-infected plants for at least 2 weeks to acquire the virus before they were used to inoculate field pea and lentil plants in the virus-treated plots in the field experiments.

### Inoculation of virus-infected plots with TuYV

**Figure 1:**
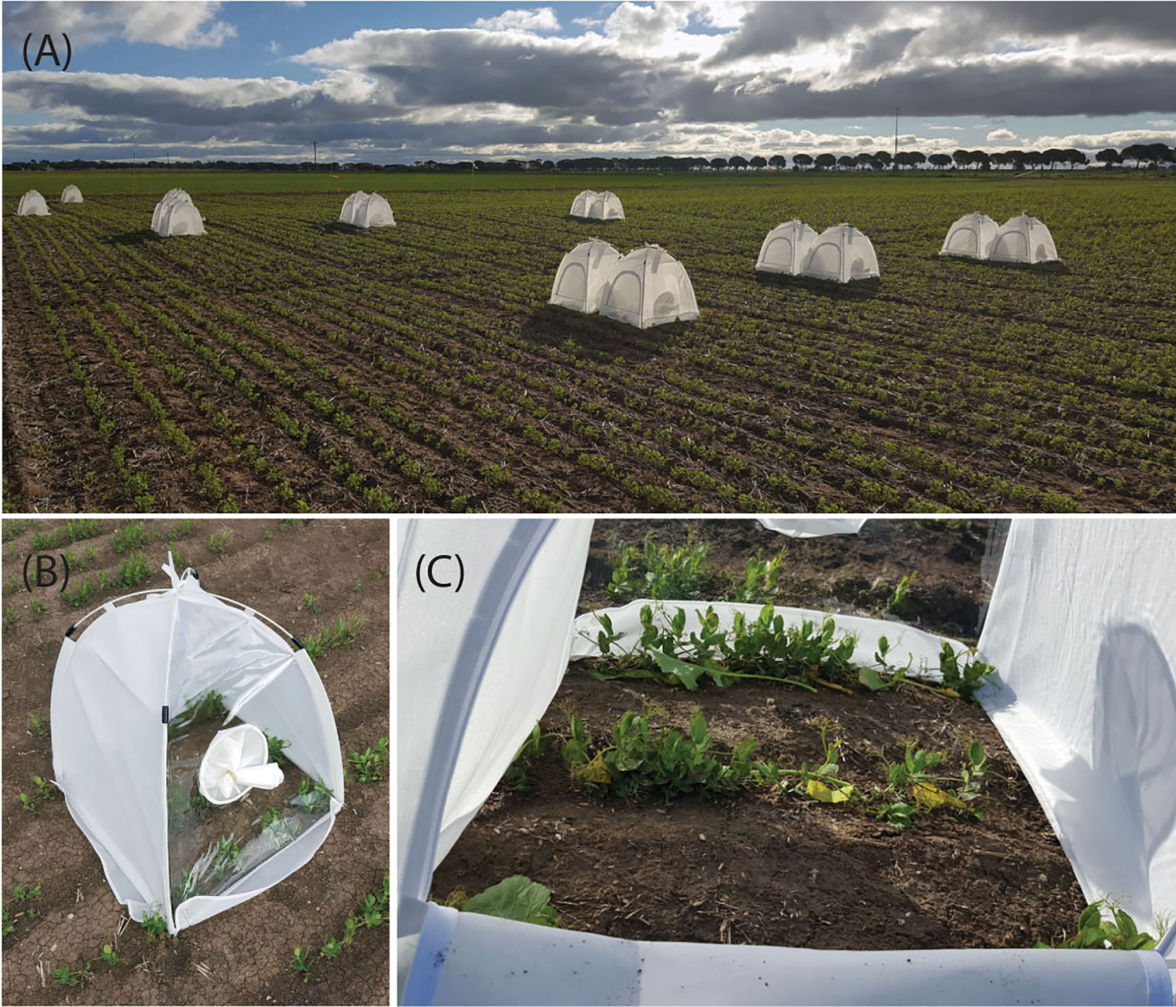
Insect cages covering experimental plots during inoculation with turnip yellows virus (TuYV) using viruliferous aphids (*Myzus persicae*) during 2020 (**A**, two cages per plot); an insect cage covering field pea seedlings in 2018 **(B)**; field pea plants covered by an insect cage with pieces of canola plant containing viruliferous aphids (*M. persicae*) placed alongside each plant **(C)**.

In the first year of the study (2018), the two experimental treatments were: early TuYV inoculation (inoculated at the seedling stage) and a non-inoculated control treatment. In the second and third years (2019 and 2020) of the trial, the three experimental treatments were: early TuYV inoculation (inoculated at the seedling stage), late TuYV inoculation (inoculated two months after the first inoculation) and a non-inoculated control treatment (**Table 1**). Each experiment consisted of a randomised block design with 6 replicates. Each experimental plot (i.e.- control and inoculated) was covered with an insect cage (60 cm x 60 cm, **Figure 1A, B**) (BugDorm, MegaView Science, Taiwan) to contain the aphids and prevent contamination of nearby plots, then pieces of canola plant containing viruliferous green peach aphid (*M. persicae*) were placed alongside each row of plants (**Figure 1C**) in the plots selected for TuYV infection, with the aim of inoculating each plant with 5-10 viruliferous aphids. The experimental plot size was equivalent to the size of either one (60 cm x 60 cm or 60 cm x 3 rows, 0.36 m^2^) or two (120 cm x 60 cm or 120 cm x 3 rows, 0.72 m^2^) insect cages: the plot size was equivalent to one cage in the first year (2018), two cages for the first inoculation and one cage for the second inoculation in the second year (2019) and two cages for both the first and second inoculation in the third year (2020). After 1-2 weeks, the cages were removed and plants were immediately sprayed with contact and systemic insecticides, pyrethrum (Yates, active ingredient: pyrethrins) and Confidor (Bayer, active ingredient: imidacloprid), respectively.

**Table 1:** Sowing, inoculation, virus testing and harvesting dates for each experiment conducted in south-eastern Australia during 2018-2020 to examine the impacts of turnip yellows virus (TuYV) infection on grain yield of lentil and field pea.

	2018	2019	2020
	Lentil		
**Sown**	25^th^ May2018	22^nd^ May 2019	25^th^ May 2020
**Inoculation 1**	2^nd^ - 17^th^ August 2018	15^th^ - 22^nd^ July 2019	15^th^ - 23^rd^ July 2020
**Inoculation 2**	No 2nd inoculation	23^rd^ - 30^th^ August 2019	17^th^ - 26^th^ August 2020
**Virus testing**	5^th^ November 2018	22^nd^ October 2019	6^th^ November 2020
**Harvest**	30^th^ November 2018	4^th^ December 2019	10^th^ December 2020
	**Field pea**		
**Sown**	25^th^ May 2018	22^nd^ May 2019	25^th^ May 2020
**Inoculation 1**	20^th^ July 2018 - 2^nd^ August 2018	22^nd^ - 30^th^ July 2019	15th - 23^rd^ July 2020
**Inoculation 2**	No 2nd inoculation	23^rd^ - 30^th^ August 2019	17^th^ - 26^th^ August 2020
**Virus testing**	5^th^ November 2018	22^nd^ October 2019	6^th^ November 2020
**Harvest**	19^th^ November 2018	3^rd^ December 2019	10^th^ December 2020

### Assessment of virus symptoms and incidence

Plant growth was regularly monitored for the presence of symptoms typical of virus infection, such as stunted or spindly growth and yellow or red leaf symptoms, by visual observation throughout each experiment. In addition, to assess the effectiveness of the inoculation method and levels of background infection in each year, 15-30 tendrils or leaf stems were randomly collected from each plot before maturity and tested for TuYV using TBIA as described previously by Freeman et al. (2013). The percentage of positive samples collected from each plot and mean incidence (i.e.- proportion of infected plants) for each treatment were then calculated.

Background virus infection was further examined by testing the plant samples for cucumber mosaic virus (CMV) in 2018-2020 and pea seed-borne mosaic virus (PSbMV) in 2019 using TBIA as described previously by Freeman et al. (2013).

### Plant growth assessments

Assessments were conducted to examine the effects of TuYV infection on plant growth during 2019 and 2020, but not during 2018.

Plant growth was assessed in the field twice during the 2019 growing season: the first assessment was done two months after the first (and one month after the second) inoculation while the second assessment was done 3.5 months after the first (and 2.5 months after the second) inoculation. Ten plants from each plot were assessed each time. To measure the ‘greenness’ (chlorophyll content) of field pea, one SPAD reading was taken from the top-most fully- extended leaf from each plant using a SPAD-502Plus chlorophyll meter (Konica Minolta, Tokyo, Japan), then the mean SPAD for each plot was calculated. In lentil, plant height was measured instead of SPAD (due to the small size of the leaves) by measuring the distance from the base of the plant to the tallest tip of the plant.

More detailed assessments of plant growth were carried out during the third year of the study (2020) for both lentil and field pea to examine the effects of TuYV infection on plant physiology that might be associated with virus-related grain yield losses. Assessments were done at three stages throughout the growing season: at the time of the first or second inoculation, 6 weeks after the first inoculation and 10 weeks after the first inoculation (which was also 5 weeks after the second inoculation). The number of plants collected from each plot for each assessment is shown in **Supplementary Table 1**. The whole “above-ground” portion of the plant was collected, placed into a plastic bag and transported to the laboratory. Assessments were carried out on each individual plant and values were then averaged to obtain a final value for each plot. The height of each plant was measured using a ruler and the mean plant height for each plot was calculated. Each plant was tested for TuYV using TBIA as described by Freeman et al. (2013). One SPAD reading was taken from the topmost fully extended leaf from each plant, and the mean SPAD for each plot was calculated. An estimation of the total leaf area for each plot was obtained by removing the leaves from all plants from each plot and measuring the leaf area using a LI-3000C Portable Leaf Area Meter with an LI-3050C Transparent Belt Conveyor Accessory (LI-COR Biosciences, Lincoln, USA). Leaves and stems were then placed into paper bags and dried in a dehydrating oven (Thermoline Scientific, NSW, Australia) at 70°C for three days, then dried leaves and stems were weighed. Specific leaf area was calculated by dividing the leaf area (cm^3^) by the dried weight of the leaves (g).

### Harvest assessments

For each experiment in each year, the above-ground portion of all plants in each plot was collected at plant maturity, placed into large paper bags, and transported to the laboratory where dried biomass was measured. All field pea pods were removed by hand, counted and then weighed before grains were removed from the pods, counted and weighed. Lentil samples were threshed using a Hans-Ulrich Hege 16 laboratory thresher (Wintersteiger, Ried im Innkreis, Austria), and grains were counted and weighed. All field pea and lentil grains were counted using a Numigral seed counter (CHOPIN Technologies, Cedex, France), and the 1,000-grain weight calculated.

### Weather and grain production data

Temperature and rainfall data for the field site were obtained from weather station number 79028, which was located adjacent to the field site, from the Bureau of Meteorology (BOM, www.bom.gov.au) and SILO (www.longpaddock.qld.gov.au/silo) websites (accessed on 15^th^ June 2021). Average annual rainfall and temperature data were used to demonstrate the variation in weather conditions at the field site in each year of the study (**Supplementary Table 2**). Long-term mean rainfall and temperature values were calculated using all available data from 1961-2020.

### Data analysis

Mean differences in grain yield (g/plot) between the inoculated and non-inoculated control treatments were calculated and the percentage difference in yield determined. It was assumed that each plot had approximately the same number of plants in 2018, however the number of plants in each plot was counted when the inoculation cages were removed in 2019 and 2020. Therefore in 2018, all parameters were calculated on a per plot basis. In 2019 and 2020, each parameter was divided by the number of plants in the plot so were calculated on a per plant basis, then that value was multiplied by the average number of plants in a plot (90 for lentil, 30 for field pea) to obtain a ‘per plot’ estimation for graphical presentation.

GenStat 14^th^ Edition (VSN International, Hemel Hempstead, UK) was used to obtain means and standard errors of the means. Data sets were tested for normality using quantile-quantile plots, then analysis of variance (ANOVA) and Tukey’s honestly significant difference (HSD) tests were carried out on non-transformed data to test for statistical significance, using R (R Foundation for Statistical Computing, Vienna, Austria); differences were considered statistically significant at p < 0.05.

## Results

**Figure 2:**
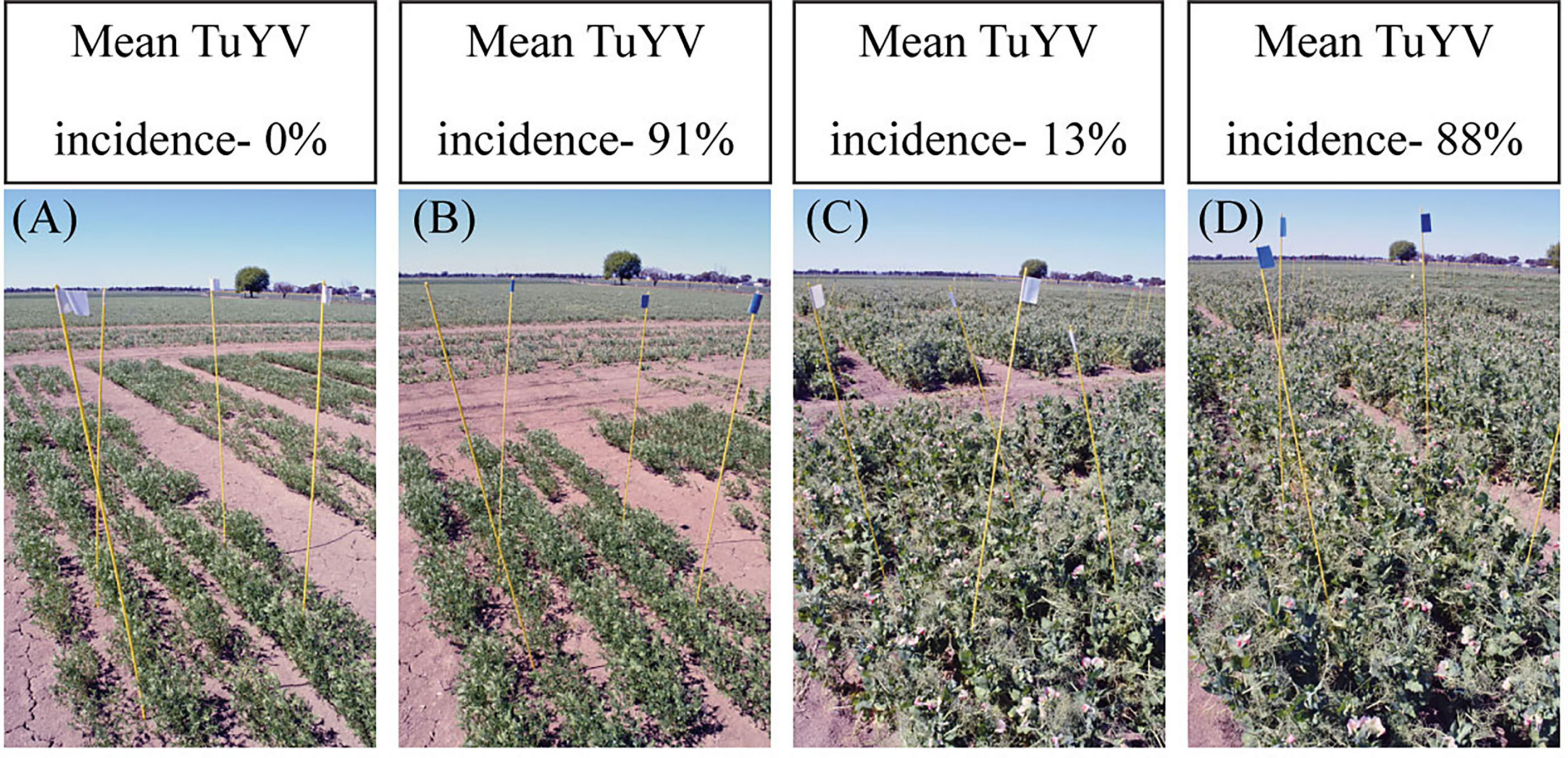
The lack of obvious symptoms of virus infection in: **(A)** non-inoculated control and **(B)** inoculated plots of lentil, and **(C)** non-inoculated control and **(D)** inoculated plots of field pea in field experiments conducted in south-eastern Australia during 2018.

During the three years of this study, six individual field experiments were conducted to quantify grain yield losses associated with TuYV infection in lentil and field pea in south-eastern Australia. Seasonal conditions varied across the three years of the study, resulting in different mean maximum temperatures and annual rainfall each year (**Supplementary Table 2**). The method used to inoculate virus-treated experimental plots was successful in each year of the study, resulting in 73-100% incidence of TuYV infection in inoculated plots and 0-18% incidence in non-inoculated control plots (**Table 2**). However, despite the TuYV infection present in the inoculated plots of both lentil and field pea in each year of the study, no typical or obvious symptoms of virus infection were observed in virus-inoculated lentil or field pea plots throughout the three years of the study (**Figure 2** and **Supplementary Figure 1**); there was no indication that the plants in the inoculated plots were infected until the plants were tested for TuYV using TBIA. CMV and PSbMV were either not detected or were detected with very low incidence (≤2%) (**Table 2**).

**Table 2:** Mean incidence (percentage of infected plants) of turnip yellows virus (TuYV), cucumber mosaic virus (CMV) and pea seed-borne mosaic virus (PSbMV) in plots of field-grown lentil and field pea for each treatment (i.e.- non-inoculated control, early TuYV infection and late TuYV infection) in field experiments conducted in south-eastern Australia during 2018-2020; virus incidence was determined using tissue blot immunoassay.

Year	Host	Treatment	Mean virus incidence (%)		
			TuYV	CMV	PSbMV
2018	Lentil	Control	0	0	-^A^
		Early TuYV	91	0	–
2018	Field pea	Control	13	0	–
		Early TuYV	88	0	–
2019	Lentil	Control	8	0	0
		Early TuYV	74	0	0
		Late TuYV	87	2	1
2019	Field pea	Control	18	0	0
		Early TuYV	83	0	0
		Late TuYV	83	0	0
2020	Lentil	Control	0	1	–
		Early TuYV	98	0	–
		Late TuYV	73	0	–
2020	Field pea	Control	11	0	–
		Early TuYV	100	0	–
		Late TuYV	94	0	–

^A^- Plant samples were not tested for PSbMV in 2018 or 2020.

### Effect of TuYV infection on grain yield

#### Lentil and field pea; 2018 (early TuYV infection and non-inoculated control)

**Figure 3:**
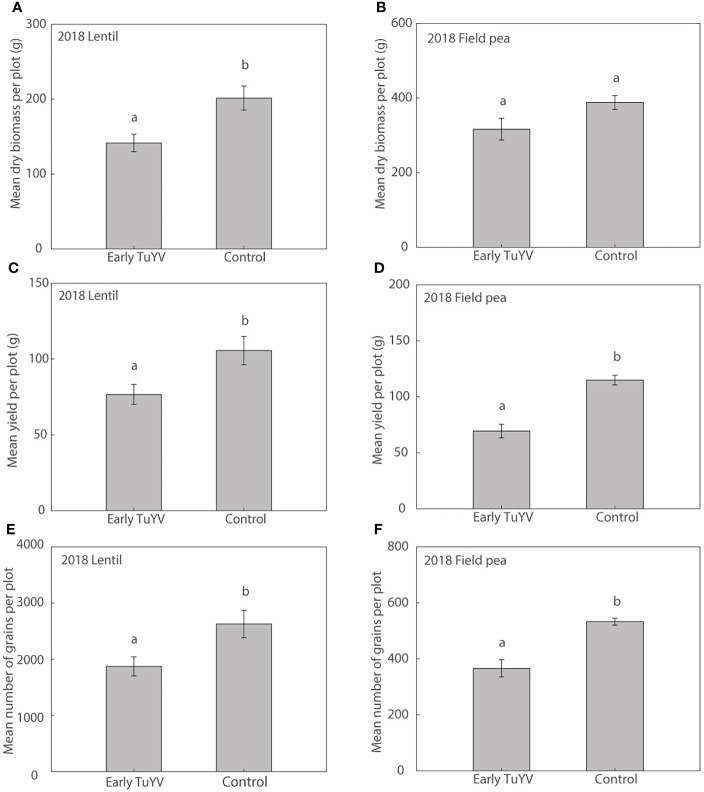
The effect of early turnip yellows virus (TuYV) infection on dry plant biomass **(A, B)**, grain yield **(C, D)** and number of grains **(E, F)** of lentil (left) and field pea (right) in field experiments (2018) in south-eastern Australia. Standard errors are represented by error bars; means with different letters are significantly different at p < 0.05 according to ANOVA.

In 2018, dry plant biomass at harvest was significantly reduced by 30% (p=0.022) in lentil (**Figure 3A**) but was not significantly affected by early TuYV infection in field pea (p=0.06, **Figure 3B**). Despite the lack of noticeable visual symptoms of virus infection, early TuYV infection significantly reduced grain yield by 28% (p = 0.030) in lentil (**Figure 3C**) and by 40% (p < 0.001) in field pea (**Figure 3D**) compared to the non-inoculated control plots. The number of grains collected per plot was also significantly reduced by early TuYV infection by 29% (p = 0.029) in lentil (**Figure 3E**) and 31% (p < 0.001) in field pea (**Figure 3F**); early TuYV infection did not have any significant effect on 1000-grain weight in lentil (p = 0.33) (**Supplementary Table 3**) but reduced 1000-grain weight by 12% (p < 0.001) in field pea (**Supplementary Table 4**). TuYV infection also significantly reduced the number of pods per plot by 30% (p = 0.001) and the weight of pods per plot by 39% (p < 0.001) in field pea, however these parameters were not measured in lentil.

Weather conditions were warm and dry in the Wimmera region in 2018 (**Supplementary Table 2**); the mean maximum temperature was 1.5°C above average while annual rainfall was 44% below average.

#### Lentil and field pea; 2019 (early TuYV infection, late TuYV infection and non-inoculated control)

**Figure 4:**
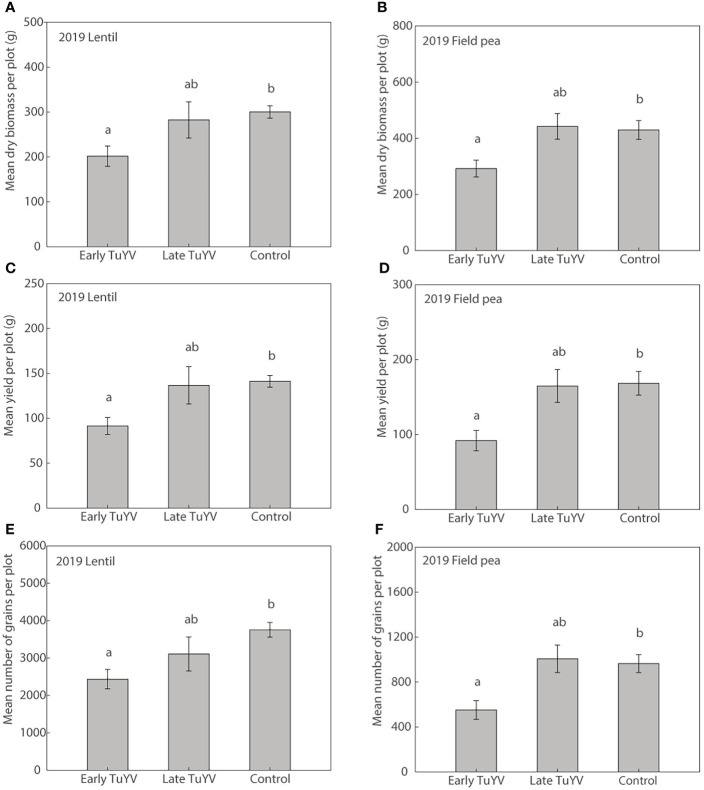
The effect of early and late turnip yellows virus (TuYV) infection on dry plant biomass **(A, B)**, grain yield **(C, D)** and number of grains **(E, F)** of lentil (left) and field pea (right) in field experiments (2019) in south-eastern Australia. Standard errors are represented by error bars; means with different letters are significantly different at p < 0.05 according to ANOVA.

In 2019, no noticeable typical symptoms of virus infection were observed in either field pea or lentil. Dry plant biomass at harvest was significantly reduced by early TuYV infection by 33% (p=0.017) in lentil (**Figure 4A**) and 32% (p=0.045) in field pea (**Figure 4B**) compared to the non-inoculated control treatment. Despite the lack of noticeable visual symptoms of virus infection, early TuYV infection significantly reduced grain yield by TuYV infection by 35% (p = 0.014) in lentil (**Figure 4C**) and 45% (p =0.017) in field pea (**Figure 4D**). The number of grains collected per plot was also significantly reduced by early TuYV infection by 35% (p = 0.009) in lentil (**Figure 4E**) and 43% (p < 0.015) in field pea (**Figure 4F**). Early TuYV infection also significantly reduced the weight of the pods by 39% (p < 0.001) and the number of pods by 37% (p = 0.034) in field pea, however these parameters were not measured in lentil. There were no significant effects of early TuYV infection on 1000-grain weight in either lentil (p = 0.21) or field pea (p = 0.60) (**Supplementary Tables 5 and 6**). Additionally, late TuYV infection did not significantly affect grain yield (**Figures 4A, B**), the number of grains (**Figures 4C, D**), dry plant biomass (**Figures 4E, F**), 1,000-grain weight in lentil (p=0.42) or field pea (p=0.32), nor did it significantly affect the weight of pods (p=0.98) or the number of pods (p=0.83) in field pea, compared to the non-inoculated control treatment (**Supplementary Tables 5 and 6**).

Weather conditions were warmer and drier than average in the Wimmera region during 2019 (**Supplementary Table 2**); the mean maximum temperature was 1.2°C above average while annual rainfall was 23% below average.

#### Lentil and field pea; 2020 (early TuYV infection, late TuYV infection and non-inoculated control)

**Figure 5:**
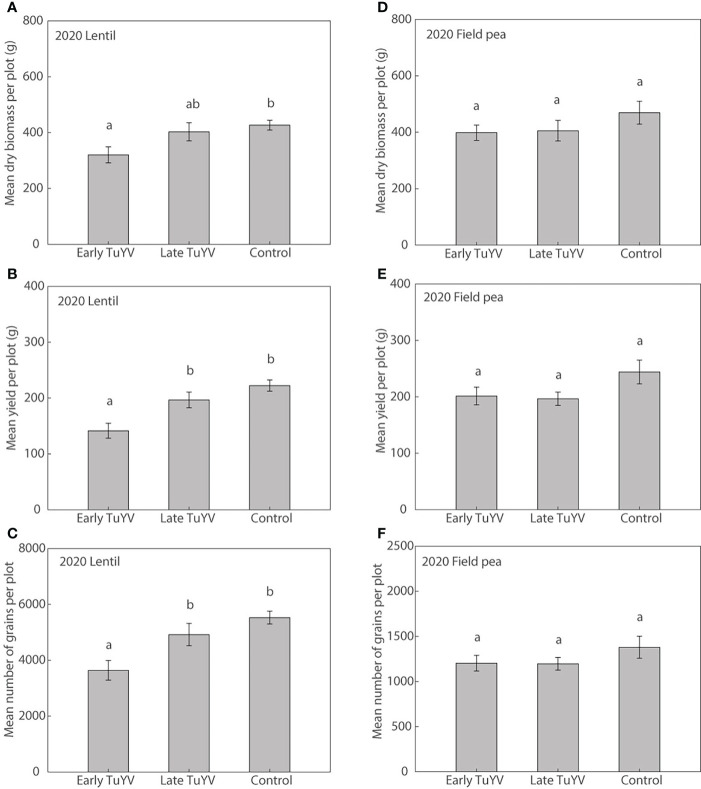
The effect of early and late turnip yellows virus (TuYV) infection on dry plant biomass **(A, D)**, grain yield **(B, E)** and number of grains **(C, F)** of lentil (left) and field pea (right) in field experiments (2020) in south-eastern Australia. Standard errors are represented by error bars; means with different letters are significantly different at p < 0.05 according to ANOVA.

In 2020, no noticeable typical symptoms of virus infection were observed in either field pea or lentil. Dry plant biomass at harvest was significantly reduced by early TuYV infection by 25% (p=0.013) in lentil (**Figure 5A**) compared to the non-inoculated control treatment. Despite the lack of noticeable visual symptoms of virus infection, early TuYV infection significantly reduced grain yield by 36% (p < 0.001, **Figure 5B**), the number of grains by 34% (p < 0.001, **Figure 5C**) but did not significantly affect 1000-grain weight (p = 0.13) in lentil when compared with the non-inoculated control treatment. Late TuYV infection did not significantly affect dry plant biomass (p = 0.77), grain yield (p = 0.31), number of grains (p = 0.35), or 1000-grain weight (p = 0.63) in lentil (**Supplementary Table 7**). However, in 2020, early TuYV infection did not significantly affect dry plant biomass (p=0.45, **Figure 5D**), grain yield (p=0.32, **Figure 5E**), number of grains (p=0.56, **Figure 5F**), weight of pods (p=0.30), number of pods (p=0.97) or 1,000-grain weight (p=0.08) in field pea (**Supplementary Table 8**). Similarly, late TuYV infection did not significantly affect dry plant biomass (p=0.52), grain yield (p=0.25) or number of grains (p=0.53), weight of pods (p=0.24) or number of pods (p=0.88) in field pea, although it did significantly reduce 1,000-grain weight by 7% (p=0.028) (**Supplementary Table 8**).

Weather conditions were close to average in the Wimmera region during 2020 (**Supplementary Table 2**); the mean maximum temperature was average while annual rainfall was 13% above average.

### Plant growth assessments

No noticeable symptoms of virus infection were observed in either lentil or field pea during the plant growth assessments that were carried out in 2019 and 2020.

In the two plant growth assessments that were done during the 2019 growing season, no significant differences were observed in chlorophyll content in field pea due to early (p = 0.10- 0.22) or late (p = 0.20- 0.99) TuYV infection in either the first or second assessment in comparison to the non-inoculated control treatment. No significant differences were observed in height in lentil due to early (p = 0.13- 0.66) or late (p = 0.45- 0.46) TuYV infection in either the first or second assessment.

In the detailed plant growth assessments that were carried out to examine the effects of TuYV infection on plant physiology, plant height was significantly reduced by 9% by TuYV infection in lentil in the later (third) assessment however no other significant differences associated with virus infection were found in lentil during the plant growth assessments (**Supplementary Table 9A**). In field pea, plant height, stem weight and whole plant weight were significantly reduced by 8%, 17% and 18%, respectively, by TuYV infection in the second assessment but were not significantly affected by virus infection in the later (third) assessment. No other significant differences associated with virus infection were found in field pea during the growth assessments (**Supplementary Table 9B**).

## Discussion

In six experiments conducted over three years, early TuYV infection resulted in grain yield losses of up to 36% in lentil and 45% in field pea, with yield losses varying between years. Despite the importance of the pulse industry in Australia and worldwide, little information is available about the impact of TuYV infection on yield in pulses. Importantly, this study also provides evidence of yield losses caused by non-symptomatic virus infection in both lentil and field pea, which has important implications for virus surveillance, epidemiology, disease progression and the development of effective virus management strategies. These experiments demonstrate the yield losses that TuYV infection can potentially cause in pulses under the field conditions that are experienced in south-eastern Australia when viruliferous aphids are abundant early in the growing season.

Early TuYV infection caused significant grain yield loss in lentil in each year of the study, with losses of 28%, 35% and 36% obtained in 2018, 2019 and 2020, respectively, which was primarily due to the presence of fewer grains, rather than smaller grains. Early TuYV infection also caused significant yield loss in field pea in the first two years of the study, with yield losses of 40% and 45% obtained in 2018 and 2019, respectively, however no significant yield loss was recorded in field pea in 2020. Although most of the yield loss observed in field pea in 2018 was due to the presence of fewer grains, a significant reduction in 1,000-grain weight also contributed to the yield loss observed in field pea in 2018. It has similarly been observed in field trials in canola that TuYV infection resulted in the production of fewer grains, however, grain weight has been shown to be either not significantly affected at all or increased as a result of TuYV infection in canola (Jay et al., 1999; Jones et al., 2007; Stevens et al., 2008). Additionally, the number of pods was reduced in field pea as a result of early TuYV infection in both 2018 and 2019, however, a similar study in canola showed that TuYV infection had no significant impact on the number of pods produced (Jay et al., 1999). Given that only one lentil and one field pea variety were evaluated in this study, these experiments should be conducted on more pulse host types and varieties to better understand the effect of TuYV infection in pulses, with a view to also identifying potential sources of virus- resistance for further investigation if different responses to TuYV infection are observed.

Even though the mean yield of the early infection treatment was 18% lower, and the late infection treatment was 20% lower than the non-inoculated control treatment in field pea in 2020, neither of these differences were statistically significant. This may indicate that the plot size was not large enough in this experiment to capture any differences statistically and that the plot size should be increased in future experiments (the average number of plants in a plot was approximately 30 for field pea and 90 for lentil). Or, given that the temperature and rainfall conditions were below average in 2018 and 2019, but average to above average in 2020, the lack of statistically significant yield loss recorded in field pea in 2020 may indeed be a true result and the relationship between environmental factors, TuYV infection, and yield loss is more complex in field pea than lentil.

Previous studies have shown that symptoms and yield losses are more severe in canola when plants were inoculated with TuYV in the early growth stages (Jones et al., 2007; Coutts et al., 2010; Congdon et al., 2020). Similarly, greater yield losses were observed in lentil and field pea with early TuYV infection compared to later infection in this study. In fact, no significant differences in yield were observed because of late infection in any of the four experiments that compared early and late TuYV infection, where the late inoculation treatment was applied 4-5 weeks after the early inoculation. Therefore, it is vital that plants are protected from virus infection early in the growing season. Although infection was due to artificial inoculation in this study, the plants were sprayed with insecticide immediately after inoculation, which not only limited virus transmission between plots but also removed the possibility that the yield losses observed were due to direct feeding damage caused by *Myzus persicae*, which Jones et al. (2007) showed could potentially cause some additional yield loss in canola. Although the inoculation method used in this study was effective, it is possible that the yield losses caused by TuYV infection might have been greater if virus incidence had reached 100% in the inoculated plots and 0% in the control plots each year. It is also possible that the incidence may have been higher if plants had been tested using a more sensitive method than TBIA, such as RT-PCR.

Despite the presence of TuYV infection in the inoculated lentil and field pea plots, and the significant yield losses that were observed because of TuYV infection, no obvious symptoms of virus infection were observed throughout the six experiments, even though the TuYV isolate used for the experiments still caused symptoms in canola. The lack of typical symptoms in lentil and field pea was so striking and unexpected in the first year of the study that it was initially assumed that the virus inoculations may not have worked. However, when plants from each plot were tested for TuYV infection by TBIA just before maturity, inoculated lentil and field pea plots were found to be infected with an average TuYV incidence of 88% and 91%, respectively, which was further followed by grain yield losses of 28% and 40% in lentil and field pea, respectively, at harvest. Overall, the two plant growth assessments that were carried out in 2019 and the detailed plant growth assessments that were conducted in 2020 provide further evidence of the lack of noticeable symptoms of virus infection field pea and lentil. The absence of visible symptoms of virus infection in field pea and lentil throughout the trial, despite the occurrence of significant yield loss, has important implications for disease surveillance and TuYV epidemiology within broad acre cropping systems, and highlights the importance of testing for virus instead of relying on the presence of visual symptoms to indicate virus presence. This is particularly important for pulse breeding programs which rely on the observation of visual symptoms alone to estimate virus presence or incidence, and therefore may potentially, and inadvertently, be selecting for symptomless virus infection that can still result in yield loss. The lack of visual symptoms of TuYV infection observed in this study may also be leading to an underestimation of the importance of this virus in pulses in Australia.

There are a number of reasons why TuYV infection may have been symptomless in lentil and field pea. Some environmental conditions can promote non-symptomatic virus infection. For instance, symptoms of viruses such as TuYV are sometimes ‘masked’ and the amount of vegetative growth and light intensity can affect symptom development (Johnstone et al., 1984). The development of plant virus symptoms can also vary with temperature (Nagamani et al., 2020). However, given that this study was conducted over 3 years where plants were exposed to a variety of temperatures and light intensity levels, and that healthy vegetative growth was observed in each year, these factors are not likely to be the main cause of the consistent lack of virus symptoms observed. Symptoms of TuYV infection in canola have also been shown to vary in severity with cultivar, with some canola cultivars appearing to be non-symptomatic when infected (Coutts et al., 2010). In this study, only one lentil and one field pea cultivar were evaluated so it is possible that the non-symptomatic infection was variety-specific, although the fact that it occurred in both the pulse varieties used in this study indicates that it is probably more than a rare occurrence. Given the diversity of TuYV isolates in Australia (Filardo et al., 2021), it is likely that the specific pulse cultivars and TuYV isolate used for these experiments contributed to the lack of visual symptoms, demonstrating that the relationships between different TuYV isolates, pulse varieties, symptom development and yield losses require further investigation.

Environmental factors play a major role in plant virus epidemiology, as the plant, virus and vector may have different optimal requirements to persist and thrive, consequently climatic conditions will influence disease expression. Climate change, i.e.: elevated carbon dioxide, increased temperature and changes to water availability, can also impact the epidemiology of plant viruses (Trębicki and Finlay, 2019; Trębicki, 2020; Jones et al., 2021). Although little is known about the effects of climate change on TuYV, in studies which investigated other vector transmitted viruses both elevated carbon dioxide and temperature increased virus titer and incidence (Nancarrow et al., 2014; Trębicki et al., 2016; Trębicki et al., 2017), and changes in plant biochemistry affected their vectors (Dáder et al., 2016; Trębicki et al., 2016; Carreras Navarro et al., 2020; Moreno-Delafuente et al., 2020). As a global pathogen, TuYV might express differently in different plants now and in the future.

In conclusion, the six field experiments carried out in this study quantified yield losses caused by TuYV infection in lentil and field pea in south-eastern Australia. Like other field studies that have examined yield losses associated with virus infection in grain crops (Monneveux et al., 1992; Graichen and Schliephake, 1999; Jay et al., 1999; Nancarrow et al., 2021), yield losses varied between years, again demonstrating the importance of examining the effects of virus infection on yield across multiple years. Importantly, no obvious symptoms of virus infection were observed in the virus-inoculated plots, with yield losses resulting from non-symptomatic TuYV infection. This observation has implications from an epidemiological, breeding and control perspective, and demonstrates how important it is that breeding programs test for virus presence instead of relying on visual symptoms to avoid inadvertently selecting for non-symptomatic virus infection that can still cause yield loss. We recommend that these experiments be conducted on more pulse host types and varieties, and against as wide a range of TuYV isolates as possible to provide a broader understanding of the relationships between different TuYV isolates and pulse varieties, symptom development and yield losses, and to identify pulse varieties that respond differently to virus infection as potential sources of virus-resistance for further investigation.

## Data availability statement

The original contributions presented in the study are included in the article/**Supplementary Material**. Further inquiries can be directed to the corresponding author.

## Author contributions

PT conceptualised the experiments; PT and NN designed the experiments; NN, PT, and MA conducted the experiments; NN and PT analysed experimental data; NN wrote the draft of the manuscript; PT, BR, GH, and MA reviewed the manuscript; NN edited the manuscript; PT, GH, and BR acquired funding and provided project administration. All authors contributed to the article and approved the submitted version.

## Funding

This research was funded by Agriculture Victoria and the Grains Research and Development Corporation (GRDC) projects DAV00129 and DJP1907-001RTX. The funders were not involved in the study design, collection, analysis, interpretation of data, the writing of this article or the decision to submit it for publication. All authors declare no other competing interests.

## Acknowledgments

The authors thank Joshua Fanning, Jon Baker, and Jordan McDonald for supplying seed, sowing the field trial and assisting with trial site maintenance, and Oscar Fung, Vy Pham, Harry Hollaway, Graham Exell and Eva Carreras Navarro for technical assistance.

## Conflict of interest

The authors declare that the research was conducted in the absence of any commercial or financial relationships that could be construed as a potential conflict of interest.

## Publisher’s note

All claims expressed in this article are solely those of the authors and do not necessarily represent those of their affiliated organizations, or those of the publisher, the editors and the reviewers. Any product that may be evaluated in this article, or claim that may be made by its manufacturer, is not guaranteed or endorsed by the publisher.
