# Supplementary material for: Symptomless turnip yellows virus infection causes grain yield loss in lentil and field pea: A three-year field study in south-eastern Australia
Source: Front Plant Sci. 2022 Nov 23;13:1049905. doi: 10.3389/fpls.2022.1049905 (PMC9727233; doi:10.3389/fpls.2022.1049905)
Supplement: Supplementary file 1 [file DataSheet_1.docx]

**Supplementary Table 1.** The number of plants collected from each inoculated and control plot to carry out the detailed plant growth assessments of lentil and field pea to examine the effects of TuYV infection on plant physiology associated with yield loss in south-eastern Australia during 2020.

| **Assessment no.** | **Date** | **Crop type** | **Number of plants collected and assessed from each plot** |
| --- | --- | --- | --- |
| 1a (at inoculation 1) | 21/07/2020 | Lentil | 1 |
|  |  | Field pea | 1 |
| 1b (at inoculation 2) | 19/08/2020 | Lentil | 1 |
|  |  | Field pea | 1 |
| 2 (6 weeks after inoculation 1) | 27/08/2020 | Lentil | 10 |
|  | 24/08/2020 | Field pea | 6 |
| 3 (10 weeks after inoculation 1 & 5 weeks after inoculation 2) | 23/09/2020 | Lentil | 10* |
|  | 21/09/2020 | Field pea | 6 |

* For lentil only during assessment 3, an additional 60 plants were collected from each treatment from the first inoculation and 30 additional plants were collected from each treatment from the second inoculation for additional bulk plant biomass measurements.

**Supplementary Table 2.** Summary of the three-monthly and annual rainfall (mm) and mean maximum temperature (°C) (three-monthly) for the years 2017-2020 and the three-monthly long-term (1961-2020) means at Longerenong, Victoria, South-Eastern Australia.

|  | **Year** | **Jan-Mar** | **Apr-Jun** | **Jul-Sep** | **Oct-Dec** | **Annual** |
| --- | --- | --- | --- | --- | --- | --- |
| Rainfall (mm) | 2017 | 56 | 135 | 128 | 112 | 431 |
|  | 2018 | 24 | 82 | 79 | 42 | 227 |
|  | 2019 | 35 | 141 | 96 | 39 | 311 |
|  | 2020 | 95 | 113 | 144 | 102 | 454 |
| Long-term mean | 1961-2020 | 67 | 108 | 132 | 96 | 403 |
| Temperature (°C) | 2017 | 30.3 | 17.9 | 15.3 | 27.2 | 22.7 |
|  | 2018 | 30.6 | 19 | 16 | 26.7 | 23.1 |
|  | 2019 | 31.2 | 18.5 | 15 | 26.5 | 22.8 |
|  | 2020 | 28.5 | 17.1 | 15.5 | 25.4 | 21.6 |
| Long-term mean | 1961-2020 | 28.9 | 17.8 | 15.2 | 24.6 | 21.6 |


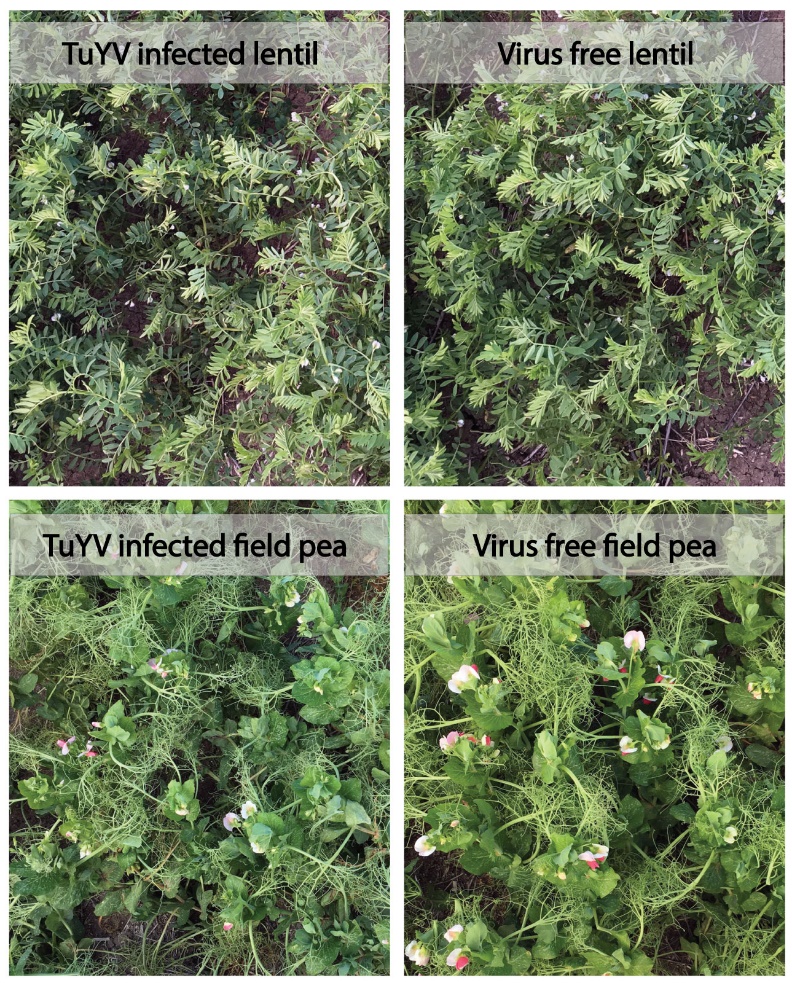


**Supplementary Figure 1.** The lack of obvious symptoms of virus infection in lentil and field pea plots infected with turnip yellows virus (TuYV), in comparison to non-inoculated plots in field experiments conducted in south-eastern Australia during 2018.

**Supplementary Table 3.** Percent change associated with the early TuYV inoculation treatment in comparison to the control treatment for grain and plant parameters measured in lentil during 2018, and P values calculated according to ANOVA for each comparison between the two treatments. Significant P values (≤0.05) are given in bold.

| **Lentil 2018** | **Percent change** | **P values** |
| --- | --- | --- |
|  | Early TuYV vs Control | |
| Dry plant biomass (g) | -30 | **0.022** |
| Grain yield (g) | -28 | **0.030** |
| Number of grains | -29 | **0.029** |
| 1,000-grain weight (g) | 2 | 0.33 |

**Supplementary Table 4.** Percent change associated with the early TuYV inoculation treatment in comparison to the control treatment for grain and plant parameters measured in field pea during 2018, and P values calculated according to ANOVA for each comparison between the two treatments. Significant P values (≤0.05) are given in bold.

| **Field pea 2018** | **Percent change** | **P values** |
| --- | --- | --- |
|  | Early TuYV vs Control | |
| Dry plant biomass (g) | -18 | 0.06 |
| Grain yield (g) | -40 | **< 0.001** |
| Number of grains | -31 | **< 0.001** |
| 1,000-grain weight (g) | -12 | **< 0.001** |
| Number of pods | -30 | **0.001** |
| Weight of pods (g) | -39 | **< 0.001** |

**Supplementary Table 5.** (A) Percent change associated with early and late TuYV inoculation treatments in comparison to the control treatment for grain and plant parameters measured in lentil during 2019. (B) The P values calculated according to ANOVA for each comparison between the three treatments (early TuYV, late TuYV and control) for parameters measured in lentil during 2019. Significant P values (≤0.05) are given in bold.

| **Lentil 2019 (A)** | **Percent change** | | |
| --- | --- | --- | --- |
|  | Early TuYV vs Control | Late TuYV vs Control | Early TuYV vs Late TuYV |
| Dry plant biomass (g) | -33 | -6 | -29 |
| Grain yield (g) | -35 | -3 | -33 |
| Number of grains | -35 | 17 | -22 |
| 1,000-grain weight (g) | 13 | 9 | 3 |
|  |  |  |  |
|  |  |  |  |
| **Lentil 2019 (B)** | **P values** | | |
|  | Early TuYV vs Control | Late TuYV vs Control | Early TuYV vs Late TuYV |
| Dry plant biomass (g) | **0.017** | 0.85 | 0.10 |
| Grain yield (g) | **0.014** | 0.96 | 0.06 |
| Number of grains | **0.009** | 0.26 | 0.33 |
| 1,000-grain weight (g) | 0.21 | 0.42 | 0.91 |

**Supplementary Table 6.** (A) Percent change associated with early and late TuYV inoculation treatments in comparison to the control treatment for grain and plant parameters measured in field pea during 2019. (B) The P values calculated according to ANOVA for each comparison between the three treatments (early TuYV, late TuYV and control) for parameters measured in field pea during 2019. Significant P values (≤0.05) are given in bold.

| **Field pea 2019 (A)** | **Percent change** | | |
| --- | --- | --- | --- |
|  | Early TuYV vs Control | Late TuYV vs Control | Early TuYV vs Late TuYV |
| Dry plant biomass (g) | -32 | 3 | 34 |
| Grain yield (g) | -45 | -2 | -44 |
| Number of grains | -43 | 4 | -45 |
| 1,000-grain weight (g) | -4 | -6 | 2 |
| Number of pods | -37 | 8 | -42 |
| Weight of pods (g) | -46 | -3 | -44 |
|  |  |  |  |
|  |  |  |  |
| **Field pea 2019 (B)** | **P values** | | |
|  | Early TuYV vs Control | Late TuYV vs Control | Early TuYV vs Late TuYV |
| Dry plant biomass (g) | **0.045** | 0.97 | 0.06 |
| Grain yield (g) | **0.017** | 0.99 | 0.05 |
| Number of grains | **0.015** | 0.95 | **0.021** |
| 1,000-grain weight (g) | 0.60 | 0.32 | 0.90 |
| Number of pods | **0.034** | 0.83 | **0.025** |
| Weight of pods (g) | **<0.001** | 0.98 | **0.045** |

**Supplementary Table 7.** (A) Percent change associated with early and late TuYV inoculation treatments in comparison to the control treatment for grain and plant parameters measured in lentil during 2020. (B) The P values calculated according to ANOVA for each comparison between the three treatments (early TuYV, late TuYV and control) for parameters measured in lentil during 2020. Significant P values (≤0.05) are given in bold.

| **Lentil 2020 (A)** | **Percent change** | | |
| --- | --- | --- | --- |
|  | Early TuYV vs Control | Late TuYV vs Control | Early TuYV vs Late TuYV |
| Dry plant biomass (g) | -25 | -6 | -21 |
| Grain yield (g) | -36 | -12 | -28 |
| Number of grains | -34 | 11 | 26 |
| 1,000-grain weight (g) | -5 | -2 | -3 |
|  |  |  |  |
|  |  |  |  |
| **Lentil 2020 (B)** | **P values** | | |
|  | Early TuYV vs Control | Late TuYV vs Control | Early TuYV vs Late TuYV |
| Dry plant biomass (g) | **0.013** | 0.76 | 0.11 |
| Grain yield (g) | **<0.001** | 0.31 | **0.029** |
| Number of grains | **<0.001** | 0.35 | **0.044** |
| 1,000-grain weight (g) | 0.13 | 0.63 | 0.61 |

**Supplementary Table 8.** (A) Percent change associated with early and late TuYV inoculation treatments in comparison to the control treatment for grain and plant parameters measured in field pea during 2020. (B) The P values calculated according to ANOVA for each comparison between the three treatments (early TuYV, late TuYV and control) for parameters measured in field pea during 2020. Significant P values (≤0.05) are given in bold.

| **Field pea 2020 (A)** | **Percent change** | | |
| --- | --- | --- | --- |
|  | Early TuYV vs Control | Late TuYV vs Control | Early TuYV vs Late TuYV |
| Dry plant biomass (g) | -15 | -14 | -2 |
| Grain yield (g) | -18 | -20 | -2 |
| Number of grains | -13 | -13 | 1 |
| 1,000-grain weight (g) | -6 | -7 | 1 |
| Number of pods | -3 | -6 | 3 |
| Weight of pods (g) | -18 | -20 | 2 |
|  |  |  |  |
|  |  |  |  |
| **Field pea 2020 (B)** | **P values** | | |
|  | Early TuYV vs Control | Late TuYV vs Control | Early TuYV vs Late TuYV |
| Dry plant biomass (g) | 0.45 | 0.52 | 0.99 |
| Grain yield (g) | 0.32 | 0.25 | 0.99 |
| Number of grains | 0.56 | 0.53 | 0.99 |
| 1,000-grain weight (g) | 0.08 | **0.028** | 0.85 |
| Number of pods | 0.97 | 0.88 | 0.97 |
| Weight of pods (g) | 0.3 | 0.24 | 0.99 |

**Supplementary Table 9.** Plant growth assessments (plant height, chlorophyll content or ‘greenness’, dry weight of leaves, stems and whole plant, dry leaf/stem ratio, leaf area and specific leaf area- ratio of leaf area/ dry weight of leaves) for lentil (A) and field pea (B) in field trials conducted in Longerenong, Victoria during 2020. Assessments were done at the time of the first (assessment 1a) and second (assessment 1b) inoculations, 6 weeks after the first inoculation (assessment 2) and 10 weeks after the first inoculation/5 weeks after the second inoculation (assessment 3).

| **A. LENTIL** | | **Assessment 1a** | | | |  | | **Assessment 1b** | | | |  | | **Assessment 2** | | | |  | | **Assessment 3** | | | | |  |  |
| --- | --- | --- | --- | --- | --- | --- | --- | --- | --- | --- | --- | --- | --- | --- | --- | --- | --- | --- | --- | --- | --- | --- | --- | --- | --- | --- |
|  |  | Control | | Early TuYV infection | |  | | Control | | Late TuYV infection | |  | | Control | | Early TuYV infection | |  | | Control | | Early TuYV infection | | Late TuYV infection | | |
| Height (cm) | | 9.8 | | 9.6 *ns* | |  | | 16.5 | | 16.3 *ns* | |  | | 18.2 | | 16.9 *ns* | |  | | 36.4 | | 32.8 * | | 35.8 *ns* | | |
| SPAD ("greenness") | | 59.3 | | 58.1 *ns* | |  | | 50.0 | | 50.2 *ns* | |  | | 48.0 | | 46.1 *ns* | |  | | 50.0 | | 49.1 *ns* | | 51.8 *ns* | | |
| Dry weight of leaves (g) | | 0.04 | | 0.04 *ns* | |  | | 0.2 | | 0.2 *ns* | |  | | 2.2 | | 1.8 *ns* | |  | | 9.3 | | 7.5 *ns* | | 7.7 *ns* | | |
| Dry weight of stems (g) | | 0.1 | | 0.1 *ns* | |  | | 0.2 | | 0.2 *ns* | |  | | 1.8 | | 1.5 *ns* | |  | | 12.1 | | 10.0 *ns* | | 10.2 *ns* | | |
| Dry weight of whole plant (g) | | 0.1 | | 0.1 *ns* | |  | | 0.3 | | 0.3 *ns* | |  | | 4.0 | | 3.4 *ns* | |  | | 21.3 | | 17.6 *ns* | | 17.9 *ns* | | |
| Ratio of leaves/stems | | 2.0 | | 2.0 *ns* | |  | | 0.9 | | 1.0 *ns* | |  | | 1.2 | | 1.2 *ns* | |  | | 0.8 | | 0.8 *ns* | | 0.8 *ns* | | |
| Leaf area (cm^2^) | | 8.9 | | 8.0 *ns* | |  | | 35.1 | | 35.0 *ns* | |  | | 445.7 | | 369.4 *ns* | |  | | 2054.5 | | 1758.6 *ns* | | 1718.0 *ns* | | |
| Specific leaf area (cm^2^/g) | | 203.8 | | 211.4 *ns* | |  | | 220.0 | | 223.5 *ns* | |  | | 204.6 | | 202.6 *ns* | |  | | 221.1 | | 191.5 *ns* | | 222.1 *ns* | | |
| TuYV incidence (%) | | 0 | | 0 | |  | | 0 | | 0 | |  | | 0 | | 53 | |  | | 0 | | 98 | | 73 | | |
| **B. FIELD PEA** | **Assessment 1a** | | | |  | | **Assessment 1b** | | | |  | | **Assessment 2** | | | |  | | **Assessment 3** | | | | | | |  |
|  | Control | | Early TuYV infection | |  | | Control | | Late TuYV infection | |  | | Control | | Early TuYV infection | |  | | Control | | Early TuYV infection | | Late TuYV infection | | |  |
| Height (cm) | 11.8 | | 11.9 *ns* | |  | | 24.3 | | 25.5 *ns* | |  | | 26.5 | | 24.4 * | |  | | 53.8 | | 48.8 *ns* | | 54.9 *ns* | | |  |
| SPAD ("greenness") | 40.1 | | 38.9 *ns* | |  | | 37.0 | | 35.8 *ns* | |  | | 32.8 | | 33.8 *ns* | |  | | 39.2 | | 39.3 *ns* | | 37.3 *ns* | | |  |
| Dry weight of leaves (g) | 0.1 | | 0.1 *ns* | |  | | 0.3 | | 0.4 *ns* | |  | | 2.8 | | 2.3 *ns* | |  | | 8.9 | | 8.2 *ns* | | 8.1 *ns* | | |  |
| Dry weight of stems (g) | 0.1 | | 0.1 *ns* | |  | | 0.5 | | 0.6 *ns* | |  | | 4.7 | | 3.9 * | |  | | 21.8 | | 20.4 *ns* | | 19.9 *ns* | | |  |
| Dry weight of whole plant (g) | 0.2 | | 0.2 *ns* | |  | | 0.8 | | 1.0 *ns* | |  | | 7.6 | | 6.2 * | |  | | 30.7 | | 28.6 *ns* | | 28.0 *ns* | | |  |
| Ratio of leaves/stems | 1.2 | | 1.0 *ns* | |  | | 0.6 | | 0.6 *ns* | |  | | 0.6 | | 0.6 *ns* | |  | | 0.4 | | 0.4 *ns* | | 0.4 *ns* | | |  |
| Leaf area (cm^2^) | 24.9 | | 28.6 *ns* | |  | | 86.8 | | 111.0 *ns* | |  | | 867.2 | | 699.6 *ns* | |  | | 2702.5 | | 2419.8 *ns* | | 2491.9 *ns* | | |  |
| Specific leaf area (cm^2^/g) | 250.0 | | 283.1 *ns* | |  | | 291.1 | | 280.4 *ns* | |  | | 306.1 | | 302.4 *ns* | |  | | 304.9 | | 297.0 *ns* | | 308.9 *ns* | | |  |
| TuYV incidence (%) | 0 | | 0 | |  | | 0 | | 0 | |  | | 0 | | 100 | |  | | 11 | | 100 | | 94 | | |  |

* denotes statistically significant differences and ns denotes non- statistically significant differences at p < 0.05 according to ANOVA when the inoculated treatments were compared to the control treatment.
